# Neurosteroids as Selective Inhibitors of Glycine Receptor Activity: Structure-Activity Relationship Study on Endogenous Androstanes and Androstenes

**DOI:** 10.3389/fnmol.2020.00044

**Published:** 2020-03-20

**Authors:** Julia V. Bukanova, Elena I. Solntseva, Eva Kudova

**Affiliations:** ^1^Research Center of Neurology, Moscow, Russia; ^2^Institute of Organic Chemistry and Biochemistry, Czech Academy of Sciences, Prague, Czechia

**Keywords:** neurosteroid, GABA receptor, glycine receptor, androstane, androstene, structure-activity relationship

## Abstract

The ability of androstane and androstene neurosteroids with modifications at C-17, C-5, and C-3 (compounds **1**-**9**) to influence the functional activity of inhibitory glycine and γ-aminobutyric acid (GABA) receptors was estimated. The glycine- and GABA-induced chloride current (*I*_Gly_ and *I*_GABA_) were measured in isolated pyramidal neurons of the rat hippocampus and isolated rat cerebellar Purkinje cells, correspondingly, using the patch-clamp technique. Our results demonstrate that all the nine neurosteroids display similar biological activity, namely, they strongly inhibited *I*_Gly_ and weakly inhibited *I*_GABA_. The threshold concentration of neurosteroids inducing effects on *I*_Gly_ was 0.1 μM, and for effects on *I*_GABA_ was 10–50 μM. Moreover, our compounds accelerated desensitization of the *I*_Gly_ with the IC_50_ values varying from 0.12 to 0.49 μM and decreased the peak amplitude with IC_50_ values varying from 16 to 22 μM. Interestingly, our study revealed that only compounds **4** (epiandrosterone) and **8** (dehydroepiandrosterone) were able to cause a significant change in *I*_GABA_ in 10 μM concentration. Moreover, compounds **3** (testosterone), **5** (epitestosterone), **6** (dihydroandrostenedione), and **9** (etiocholanedione) did not modulate *I*_GABA_ up to the concentration of 50 μM. Thus, we conclude that compounds **3**, **5**, **6**, and **9** may be identified as selective modulators of *I*_Gly_. Our results offer new avenues of investigation in the field of drug-like selective modulators of *I*_Gly_.

## Introduction

γ-Aminobutyric acid receptors type A and glycine receptor (GABA_A_R and GlyR) channels are the major inhibitory ligand-gated ion channels of the central nervous system which mediate both fast synaptic and tonic extrasynaptic inhibition (Lynch, 2009; Ziegler et al., 2009; Yevenes and Zeilhofer, 2011). Disturbance of functional activity of GlyRs and GABA_A_Rs underlies many neurological disorders. Dysfunction of GABA_A_Rs leads to channelopathies associated with epilepsy, insomnia, anxiety, and chronic pain (Möhler, 2006). Malfunctions of GlyR have been linked to a range of neurological disorders caused by mutations in genes which encode GlyR subunits, including hyperekplexia (mutations in the GlyR α1-subunit gene) (Lynch, 2004) or autism (mutations in the human GlyR α2-subunit gene) (Dougherty et al., 2013; Zhang et al., 2017). Finally, the α3 GlyRs have emerged as a promising therapeutic target for chronic pain, as the selective enhancement of the magnitude of the α3 GlyR current has been shown to exhibit analgesic effects in animal models of inflammatory pain (Lynch et al., 2017). In summary, diminished glycinergic inhibition (e.g., hyperekplexia, autism) would benefit most from facilitated glycinergic inhibition, through positive allosteric GlyR modulators. Interestingly, GlyRs modulation also plays a crucial role in synaptogenesis (Ganser and Dallman, 2009), neurite outgrowth (Tapia et al., 2000), or produces neuroprotection against metabolic stress such as oxygen/glucose deprivation (Tanabe et al., 2010). Given these considerations, GlyR-modulating compounds offer great potential for research on novel drug-like compounds.

**FIGURE 1:**
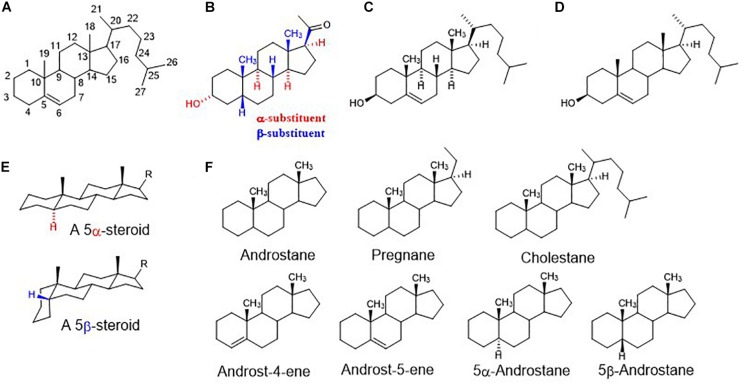
**(A)** Steroid numbering and ring letters; **(B)** schematic orientation of substituents. When the rings of a steroid are denoted as projections onto the plane of the paper, the α-substituent (hashed bond) lies below and the β-substituent (bold bond) lies above the plane of the paper; **(C)** explicitly written configuration for all sterocenters of cholesterol; **(D)** unless implied or stated to the contrary in figures and schemes, the stereochemistry of steroid molecule is simplified. Depicted structure implies that atoms or groups attached at the bridgehead positions 8, 9, 14, and 17 are oriented as shown in formula C (8β,9α,14α). Angular methyles (CH_3_) at positions 10, 13 are omitted and shown only as bold bonds; **(E)** a perspective representation of planar 5α-steroid and a bent molecule of 5β-steroid; **(F)** fundamental names of steroid skeletons relevant to this paper.

**FIGURE 2:**
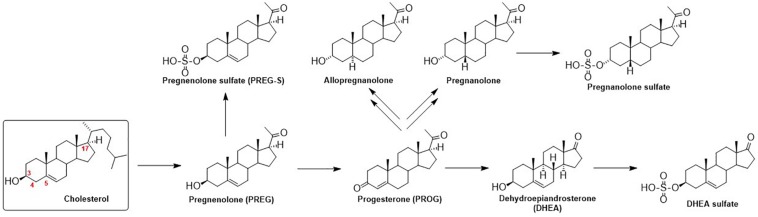
Schematic illustration of neurosteroid biosynthesis.

The function of GlyRs can be modulated by various ligands, including neurosteroids (NS). Neurosteroids are compounds that accumulate in the nervous system independently of the steroidogenic endocrine glands and which can be synthesized *de novo* in the nervous system from cholesterol or other steroidal precursors imported from peripheral sources (Baulieu, 1998). The steroid numbering, ring letters, stereochemistry and nomenclature is summarized in Figure 1. The biosynthetic pathway (Do Rego et al., 2009) of NS (Figure 2) is triggered by the conversion of cholesterol to pregnenolone (PREG). Then, PREG is converted to progesterone (PROG) and dehydroepiandrosterone (DHEA). Subsequently, PROG is metabolized to 5α- or 5β-dihydroprogesterone, followed by their reduction to 3α-hydroxy-5α-pregnan-20-one (allopregnanolone) or 3α-hydroxy-5β-pregnan-20-one (pregnanolone).

These compounds and their synthetic analogs are mainly known as potent modulators of GABA_A_Rs (Chen et al., 2019) and *N*-methyl-*D*-aspartate receptors (NMDARs) (Burnell et al., 2019), respectively. Neurosteroids and their synthetic analogs (neuroactive steroids, NAS) have been extensively studied during last three decades as they modify neuronal activity and thus brain function via a fast, non-genomic action (Rebas et al., 2017), by acting as allosteric modulators of various ligand-gated ion channels, including GABA_A_R and GlyR. In brief, NS and NAS are effective modulators of GABA_A_R-induced chloride current (I_GABA_) and their modulatory action is dependent on their structure and subtype (for a review, see: Majewska et al., 1988; Wu et al., 1990; Belelli and Lambert, 2005; Korinek et al., 2011; King, 2013; Zorumski et al., 2013). Those that potentiate GABA activity are termed as “potentiating NS” and these include, e.g., allopregnanolone (3α-hydroxy-5α-pregnan-20-one) or pregnanolone (3α-hydroxy-5β-pregnan-20-one) (Park-Chung et al., 1999). The α-configuration at C-3 is extremely important for potentiating steroids, contrasting with a relatively vague requirement for a 3α/3β-configuration for “inhibitory NS” that are referred to as those that antagonize *I*_GABA_ (Park-Chung et al., 1999). The inhibitory NS incorporate mainly a subclass known as the C-3 sulfated steroids (e.g., pregnenolone sulfate and DHEA sulfate) (Gibbs et al., 2006) or the C-3 hemiester steroids (e.g., pregnanolone hemisuccinate) (Seljeset et al., 2015), although C-3 negative charge is not obligatory for the inhibition (e.g., DHEA). The relevance of configuration or double bond at C-5 for the potentiation/inhibitory action is driven by its combination with α/β-configuration at C-3 (Park-Chung et al., 1999) that define a planar or “bent-shape” of the molecule (Figure 1E). Interestingly, the nature of the group at C-17, concerning inhibition, is less stringent given that 17-acetyl, 17-acetoxy, and 17-keto groups substituted onto a 3β-hydroxy-androst-5-ene retain similar inhibitory activities. On the other hand, 17-acetyl, 17-acetoxy, 17-hydroxyl or 17-keto groups substituted onto a 3α-hydroxy-5α-androstane exhibit markedly various enhancement of *I*_GABA_ varying up to 9-folds (Park-Chung et al., 1999). For example, the reduction of the C-20 ketone of 3α-hydroxy-5α-pregnane-20-one to its 20α-hydroxy analog greatly decreases the efficacy of potentiation 166% vs. 1373%.

The GlyR-induced chloride current (*I*_Gly_) has been also shown to be modulated by NS, but the data on potencies are rather limited to compounds with a pregnane skeleton (Figure 1F). Allopregnanolone (Figure 2) enhanced the glycine-induced current of native or recombinant receptors (Weir et al., 2004; Jiang et al., 2006), while Fodor et al. (2006) showed that micromolar concentrations of allopregnanolone blocked GlyRs of native cells. These variances may be ascribed to the difference between neuronal and recombinant GlyRs (Kung et al., 2001). Next, pregnanolone (Figure 2) proved to be an inhibitor of both α1 GlyRs and native cells (Weir et al., 2004; Fodor et al., 2006; Jiang et al., 2006). Finally, 3β-hydroxy-5α-pregnan-20-one and 3β-hydroxy-5β-pregnan-20-one were demonstrated as inactive on both neurons and recombinant α1 receptors (Wu et al., 1990; Weir et al., 2004). Interestingly, PROG exhibited incomplete and non-competitive inhibition of GlyR currents in contrast to the full and competitive inhibition by its sulfated analog (PREG-S) of chick spinal cord (Wu et al., 1997) and selectively inhibited embryonic α2 GlyRs, with no effect on α1 and α1β GlyRs (Maksay et al., 2001). To date, only three androstane compounds were tested – DHEA sulfate and 3β-hydroxy-5α-androstan-17-one, and 3α-hydroxy-5α-androstan-17-one inhibited *I*_Gly_ currents in micromolar range on recombinant α1 receptors (Maksay et al., 2001). As such, the biological potential of androstane and androstene skeletons (Figure 1F) concerning their effect on GlyR remains unknown.

In our previous work, a series of pregnanolone derivatives (modulators of NMDA receptors) displayed the effects on the *I*_GABA_ and *I*_Gly_ in rat pyramidal hippocampal neurons (Bukanova et al., 2018). Interestingly, we demonstrated that the nature of the substituent at C-3 defines the positive or negative character of *I*_GABA_. Indeed, pregnanolone glutamate was found to potentiate *I*_GABA_, while pregnanolone hemisuccinate and pregnanolone hemipimelate inhibited *I*_GABA_, and all three steroids inhibited *I*_Gly_. The conversion of the 5β-pregnanolone skeleton into an 5β-androstane skeleton, an analog that lacks the C-17 acetyl moiety, eliminated the effects on both GABA_A_Rs and GlyRs.

As mentioned previously, the modulatory effect of NS on GABA_A_Rs or GlyRs is a relevant avenue of investigation in neuropharmacology. To understand the structure-activity relationship of NS on *I*_GABA_ and *I*_Gly_, further structure-activity relationship studies (SAR) are required. In the present study, we examine the effects of a series of endogenous NS on the GABA- and Gly-induced current in voltage-clamped rat cerebellar Purkinje cells and rat hippocampal neurons, respectively. This series contained 9 natural NS with an androstane and androstene skeleton with variable substituents at C-3, C-5, and C-17 positions (Table 1).

**TABLE 1:** Structure-activity relationship study overview for compounds **1**-**9**: their chemical names, structures, τ_des_ (*I*_Gly_ vs. *I*_GABA_) values.

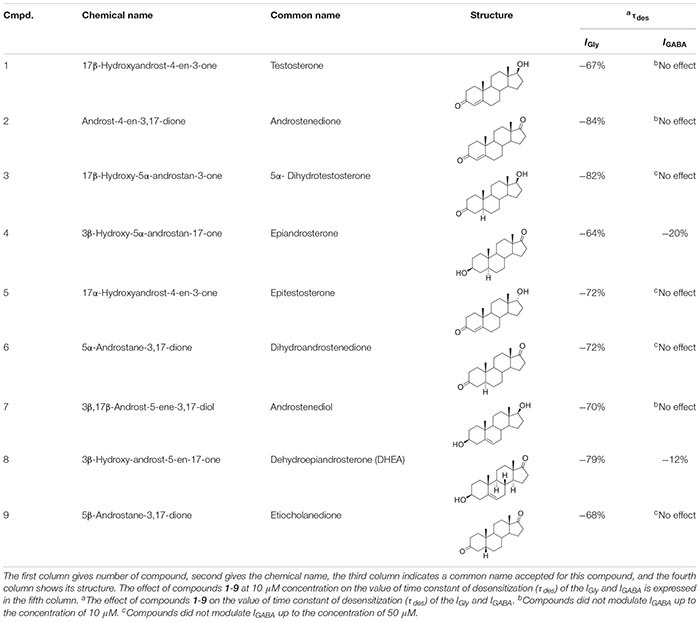

## Materials and Methods

### Cell Preparation

All experiments were conducted per the requirements of the Ministry of Public Health of the Russian Federation and were consistent with the EU directive for Use of Experimental Animals of the European Community. The study was approved by the Ethics Committee of the Scientific Center of Neurology, Protocol No. 2-5/19 of 02.20.19. The cells were isolated from transverse hippocampal slices as described in detail elsewhere (Vorobjev, 1991). Briefly, the slices (200–500 μm) of Wistar rats (11–14 days of age) hippocampus or cerebellum were incubated at room temperature for at least 2 h in a solution containing the following components (in mM): 124 NaCl, 3 KCl, 2 CaCl_2_, 2 MgSO_4_, 25 NaHCO_3_, 1.3 NaH_2_PO_4_, 10 D-glucose, pH 7.4. The saline was continuously stirred and bubbled with carbogen (95% 0_2_ + 5% CO_2_). Single pyramidal neurons from the hippocampal CA3 area or Purkinje cells from sagittal slices of the cerebellum were isolated by a vibrating fused glass pipette with a spherical tip.

### Current Recordings

Isolated neurons were patch clamped and then lifted into the outflow of the control bath solution. Bath solution flowed through a tube with a diameter of 1.5 mm at a speed of 0.6 ml/min. The substances were applied through glass capillary, 0.1 mm in diameter, which could be rapidly displaced laterally (Vorobjev et al., 1996). A fast perfusion technique allows a complete exchange of external solution surrounding a neuron within 20 ms. Glycine-activated currents (*I*_Gly_) and GABA-activated currents (*I*_GABA_) in isolated neurons were induced by a step application of agonist for 600–1000 ms with 30–40 s intervals. Transmembrane currents were recorded using a conventional patch-clamp technique in the whole-cell configuration. Patch-clamp electrodes had a tip resistance of ∼2 MΩ. The solution in the recording pipette contained the following (in mM): 40 CsF, 100 CsCl, 0.5 CaCl_2_, 5 EGTA, 3 MgCl_2_, 4 NaATP, 5 HEPES, pH 7.3. The composition of the extracellular solution was as follows (in mM): 140 NaCl, 3 KCl, 3 CaCl_2_, 3 MgCl_2_, 10 D-glucose, 10 HEPES hemisodium, and pH 7.4. Recording of the currents was performed using EPC7 patch-clamp amplifier (HEKA Electronik, Germany). The holding potential was maintained at −70 mV. Transmembrane currents were filtered at 3 kHz, stored and analyzed with IBM-PC computer, using homemade software.

### Reagents

All the drugs used for intracellular and extracellular solutions were purchased from Sigma-Aldrich (United States). Compounds **1**-**9** are available from Sigma-Aldrich or Carbosynth under the following CAS and catalog numbers: compound **1** (Sigma, CAS 58-22-0, Cat. No. T1500), compound **2** (Sigma, CAS 63-05-8, Cat. No. 46033), compound **3** (Sigma, CAS 521-18-6, Cat. No. A8380), compound **4** (Sigma, CAS 481-29-8, Cat. No. E3375), compound **5** (Sigma, CAS 481-30-1, Cat. No. 1646031), compound **8** (CAS 53-43-0, Cat No. D4000). Compound **7** (CAS 521-17-5) was prepared by sodium borohydride reduction from compound **8** according to the literature (Liu et al., 2012). Compound **6** (CAS 846-46-8) and compound **9** (CAS 1229-12-5) were prepared by Jones oxidation from compound **5** and 3α-hydroxy-5β-androstan-17-one (Sigma, CAS 53-42-9, Cat. No. E5126), respectively, according to the literature (Katona et al., 2008). The purity of all used steroids was >95%. The tested substances were dissolved in 100% DMSO to make 10 mM stock solution, which was aliquoted and stored at −20°C. Then, drugs were dissolved in external saline to the final concentrations immediately before the experiments. The maximal percentage of solvent in the tested drug solutions was 1%. The *I*_Gly_ and *I*_GABA_ were measured in the presence of 1% DMSO (*n* = 6), and any current changes was not found under these conditions.

### Data Analysis

Statistical analysis was performed with the help of *Prism Graphpad* software. All comparisons were made with ANOVA-test using Dunnett’s multiple comparison test and Student’s unpaired *t*-test at a significance level of *p* = 0.05. *N* = 5–8 cells from 3 to 4 animals for every concentration. In results descriptions, mean and standard error of the mean (SEM) are specified. The meanings of asterisks (probability levels) in figures is the following: ^∗^*P* < 0.05, ^∗∗^*P* < 0.01. The IC_50_ values for steroids inhibition of the *I*_Gly_ and *I*_GABA_ were determined using the equation: Y = 1 – [max/(1 + (IC_50_/C)^*n*^)], where *max* is the maximum inhibition attainable, *C* is the concentration of steroid, IC_50_ is the half-maximal inhibitory concentration and *n* is the slope factor (Hill coefficient).

## Results

### Effect of Neurosteroids 1-9 on the I_Gly_ and I_GABA_

The effects of compounds **1**-**9** (Table 1) were studied at a concentration range of 0.01–100 μM on isolated rat hippocampal neurons and rat cerebellar Purkinje cells. First, the ability of steroids to affect the holding current at voltage-clamp regime was tested. We have found that compounds **1**-**9** by themselves did not cause any currents through the cell membrane (data not shown). Next, the influence of compounds **1**-**9** on glycine-activated chloride current (*I*_Gly_) and GABA-activated chloride current (*I*_GABA_) were evaluated. The experiments with *I*_Gly_ were conducted on rat hippocampal neurons, and experiments with *I*_GABA_ were conducted on rat cerebellar Purkinje cells. The *I*_Gly_ is larger in amplitude and more stable on hippocampal cells, and, conversely, the *I*_GABA_ is more convenient to study on Purkinje cells, since GABA receptors on Purkinje cells are more homogeneous (Kelley et al., 2013). Glycine (100 μM) and GABA (5 μM) were applied to the neurons through an application pipette during 600–1000 ms and compounds **1**-**9** were added to the same pipette in different concentrations (0.01–100 μM). Our experiments demonstrate that neuronal GlyRs are highly sensitive, whilst neuronal GABA_A_Rs are weakly sensitive to tested compounds **1**-**9**.

### Effects of Compounds **1-9** on the I_Gly_

**FIGURE 3:**
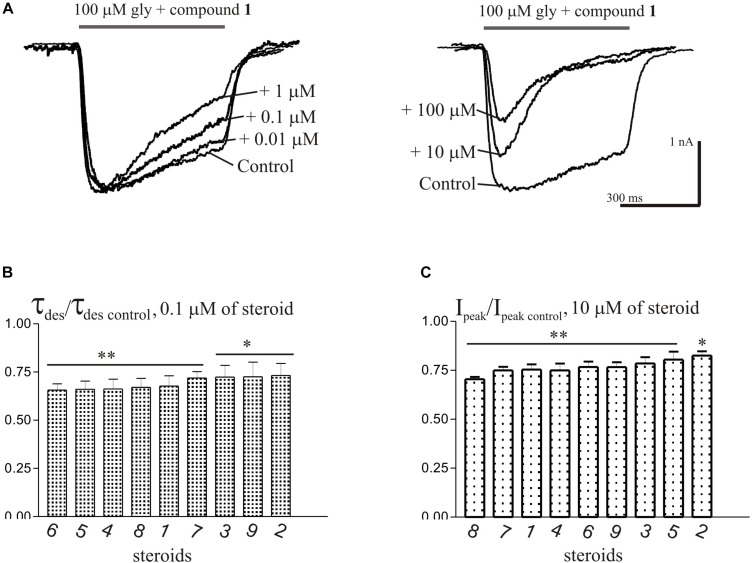
The effects of compounds **1**-**9** on *I*_Gly_ of hippocampal neurons. **(A)** Representative traces of *I*_Gly_ induced by 600 ms application of 100 μM glycine, obtained in control and the presence of 0.01, 0.1, and 1 μM (left), or 10 and 100 μM (right) of testosterone (compound **1**). **(B)** Mean ± SEM of the normalized values of the time constant of desensitization (τ_des_) of *I*_Gly_ in the presence of 0.1 μM of compounds **1**-**9**. **(C)** Mean ± SEM of the normalized values of the peak amplitude of *I*_Gly_ in the presence of 10 μM of compounds **1**-**9**. Results show greater action of NS on desensitization than on peak amplitude of *I*_Gly_. Probability levels were estimated with ANOVA-test using Dunnett’s multiple comparison test.

**FIGURE 4:**
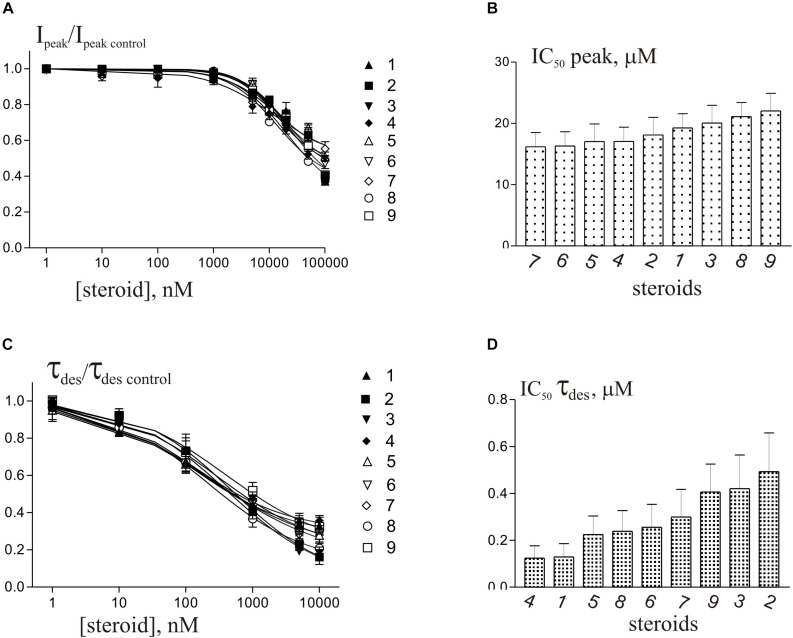
Concentration dependence of NS effects on the *I*_Gly_ of hippocampal neurons. **(A)** Concentration dependence of compounds **1**-**9** effect on the normalized peak amplitude of *I*_Gly_. Data were fitted with the Hill equation (see section Materials and Methods). **(B)** Mean± SEM of the IC_50_ values calculated for the effect of inhibition by NS of the peak amplitude of *I*_Gly_. **(C)** Concentration dependence of compounds **1**-**9** effect on the normalized τ_des_ of *I*_Gly_. Data were fitted with the Hill equation. **(D)** Mean± SEM of the IC_50_ values calculated for the effect of accelerating desensitization (decrease in the τ_des_) of *I*_Gly_ by NS.

Short (600–1000 ms) application of 100 μM glycine on pyramidal neurons of rat hippocampus evoked *I*_Gly_ which amplitude and kinetics were dependent on glycine concentration with an EC_50_ value of 90 ± 7 μM. An average value of the reversal potential of *I*_Gly_ −9.6 ± 0.8 mV matched well the chloride reversal potential calculated for the chloride concentrations used (−9.5 mV, not shown). We used agonist concentration of 100 μM that was near EC_50_, because it allow to achieve stable current with well visible and measurable either suppressive or augmenting effect. All 9 compounds caused a similar effect on the *I*_Gly_, which consisted of two components: acceleration of desensitization and decrease in peak amplitude. The effects were reversible upon washout during 1–2 min. The effect of desensitization acceleration developed at significantly lower concentrations of NS than the effect of peak amplitude suppression. Noteworthy, the threshold concentration of NS for initiating the effect of desensitization acceleration was 0.1 μM, while the threshold concentration of the same compounds for developing the effect of the peak amplitude reduction was 10 μM. A representative effect of NS on *I*_Gly_ of one cell is shown in Figure 3A. Compound **1** in low concentrations of 0.1 and 1 μM accelerated desensitization without effect on the peak amplitude, while at a concentration of 10 and 100 μM it causes two effects: acceleration of desensitization and a decrease in peak amplitude. The effects of the remaining eight NS on the *I*_Gly_ did not differ significantly from the testosterone effect (for details, see Figures 3B,C and Table 2). When co-applied with glycine, NS at concentration 0.1 μM barely affected the *I*_Gly_ peak amplitude but decreased the time constant of *I*_Gly_ desensitization (τ_des_) by 27–35% (*P* < 0.01 or *P* < 0.05). On the contrary, when applied at a concentration of 10 μM, NS accelerated desensitization by 67–82% (*P* < 0.01) and reduced the peak current amplitude by 18–25% (*P* < 0.01 or *P* < 0.05). Figure 4 shows the concentration dependence of the NS effect on the normalized peak amplitude (Figure 4A) and normalized τ_des_ of the *I*_Gly_ (Figure 4C). An increase in the concentration of NS up to 100 μM caused a decrease in the peak amplitude of the *I*_Gly_ by 45–70% with the IC_50_ values of 16–22 μM (Figure 4B and Table 3). Maximal decrease (70–90%) of the τ_des_ can be observed in the presence of 10 μM of NS. The IC_50_ values for the effect on the τ_des_ are in the range of 0.12–0.49 μM (Figures 4C,D and Table 3), which are two orders of magnitude lower than the IC_50_ values for the effect on peak amplitude.

**TABLE 2:** The inhibitory effect of the tested neurosteroids on the time constant of desensitization (τ_des_) and peak amplitude (*I*_peak_) of the *I*_Gly_. Mean ± SEM of the normalized values of the τ_des_ and *I*_peak_ of the *I*_Gly_ are shown.

**Cmpd.**	**τ_des_/τ_des control_, 0.1 μM of steroid**	***P*-value**	**n**	**I_peak_/I_peak control_ 10 μM of steroid**	***P*-value**	**n**
1	0.68 ± 0.05	0.0055	5	0.75 ± 0.03	0.0006	7
2	0.73 ± 0.06	0.0199	5	0.82 ± 0.02	0.0199	7
3	0.72 ± 0.06	0.0186	5	0.78 ± 0.03	0.0037	8
4	0.66 ± 0.05	0.0033	5	0.75 ± 0.03	0.0023	7
5	0.66 ± 0.04	0.0014	7	0.80 ± 0.04	0.0181	7
6	0.65 ± 0.03	0.0002	8	0.76 ± 0.03	0.0011	8
7	0.72 ± 0.03	0.0013	8	0.75 ± 0.02	0.0004	7
8	0.67 ± 0.05	0.0027	5	0.70 ± 0.01	0.0003	7
9	0.72 ± 0.07	0.0358	5	0.77 ± 0.03	0.0006	7

**All comparisons with control value were made with unpaired Student’s t-test. Significance level of P = 0.05. n- the number of cells used.**

**TABLE 3:** The values of the maximum inhibition attainable (*max*), the half-maximal inhibitory concentration (IC_50_) and the slope factor (Hill coefficient) for the effects of tested steroids on the peak amplitude (I_peak_) and τ_des_ of the *I*_Gly_.

**Cmpd.**	***I*_peak_**			**τ_des_**		
	***max***	**IC_50_ (μM)**	**Hill coefficient**	***max***	**IC_50_ (μM)**	**Hill coefficient**
1	0.58 ± 0.10	19.3 ± 2.3	1.1 ± 0.32	0.72 ± 0.08	0.13 ± 0.06	0.58 ± 0.20
2	0.55 ± 0.09	18.1 ± 2.9	1.1 ± 0.36	0.97 ± 0.10	0.49 ± 0.16	0.60 ± 0.11
3	0.61 ± 0.08	20.1 ± 2.8	0.86 ± 0.15	0.97 ± 0.11	0.42 ± 0.14	0.58 ± 0.13
4	0.64 ± 0.08	17.1 ± 2.3	0.97 ± 0.21	0.70 ± 0.07	0.12 ± 0.05	0.59 ± 0.16
5	0.46 ± 0.09	17.0 ± 2.9	1.2 ± 0.34	0.81 ± 0.11	0.22 ± 0.08	0.51 ± 0.15
6	0.51 ± 0.05	16.3 ± 2.4	1.2 ± 0.35	0.86 ± 0.10	0.26 ± 0.09	0.48 ± 0.10
7	0.52 ± 0.09	16.2 ± 2.3	0.71 ± 0.16	0.81 ± 0.09	0.29 ± 0.12	0.56 ± 0.13
8	0.73 ± 0.08	21.3 ± 2.2	0.92 ± 0.14	0.88 ± 0.09	0.24 ± 0.09	0.57 ± 0.14
9	0.68 ± 0.09	22.0 ± 2.8	1.0 ± 0.19	0.79 ± 0.11	0.40 ± 0.12	0.56 ± 0.14

**The fits were made to the averaged data with the fitting program.**

### Effects of Compounds **1-9** on the I_GABA_

**FIGURE 5:**
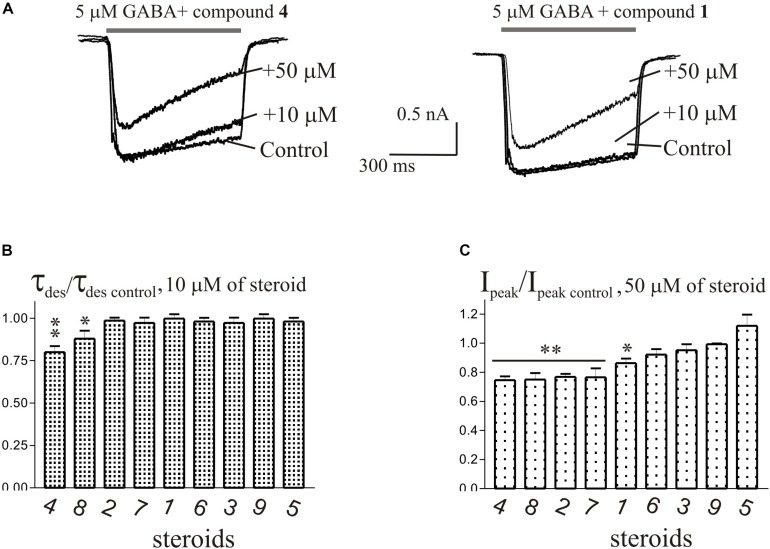
The effects of compounds **1**-**9** on *I*_GABA_ of cerebellar Purkinje cells. **(A)** Representative traces of *I*_GABA_ induced by 600 ms application of 5 μM GABA, obtained in control and the presence of 10 and 50 μM of compound **4** (epiandrosterone) (left), or compound **1** (testosterone) (right). **(B)** Mean ± SEM of the normalized values of the time constant of desensitization (τ_des_) of *I*_GABA_ in the presence of 10 μM of compounds **1**-**9**. **(C)** Mean ± SEM of the normalized values of the peak amplitude of *I*_GABA_ in the presence of 50 μM of compounds **1**-**9**. Probability levels were estimated with ANOVA-test using Dunnett’s multiple comparison test.

**FIGURE 6:**
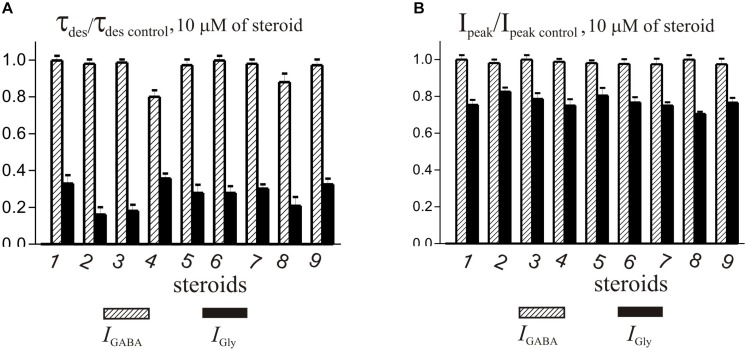
Comparison of the effects of NS on *I*_Gly_ and the *I*_GABA_. **(A)** Mean ± SEM of the normalized values of the time constant of desensitization (τ_des_) of *I*_GABA_ (shaded columns) and *I*_Gly_ (black columns) in the presence of 10 μM of compounds **1**-**9**. **(B)** Mean ± SEM of the normalized values of the peak amplitude of *I*_GABA_ (shaded columns) and *I*_Gly_ (black columns) in the presence of 10 μM of compounds **1**-**9**. Results show greater action of all nine compounds on the *I*_Gly_ than on the *I*_GABA_.

The brief application of GABA for 600–1000 ms on isolated Purkinje cells evoked a chloride current (*I*_GABA_) with an amplitude-dependent on GABA concentration with an EC_50_ value of 7.5 ± 2.9 μM. The specific antagonist of GABA_A_ receptors bicuculline (3 μM) reversibly blocked the current (data not shown), which allows us to classify the receptors as GABA_A_ type. We studied *I*_GABA_ evoked by 5 μM of GABA. Figure 5 shows the effects of NS on *I*_GABA_. Our experiments demonstrate that GABA_A_Rs are much less sensitive to the studied NS than GlyRs. The addition of compounds **1**, **2**, **3**, **5**, **7**, **9** to the applicator pipette at a concentration of 0.1–10 μM did not change either the peak amplitude or the rate of decay of *I*_GABA_. Only two out of nine compounds – compounds **4** and **8** – in 10 μM concentration were able to cause a significant change in *I*_GABA_, which consisted of the acceleration of decay (Figures 5A,B and Table 4). When the concentration of the tested compound was increased up to 50 μM, compounds **3**, **5**, **6**, and **9** remained inactive. In contrast, compounds **1**, **2**, **4**, **7**, and **8** at 50 μM concentration showed an inhibitory effect with a decrease in the peak amplitude of the current by 14–25% (*P* <0.01 or *P* < 0.05) and the acceleration of its decay by 23–45% (*P* < 0.01) (Figure 5 and Table 4). Figure 6 shows a comparison of the effects of compounds **1**-**9** on the *I*_Gly_ and the *I*_GABA_. Our results demonstrate that tested NS in the concentration of 10 μM cause strong action on *I*_Gly_ and weak action on *I*_GABA_.

**TABLE 4:** The inhibitory effect of the tested neurosteroids on the peak amplitude (*I*_peak_) and time constant of desensitization (τ_des_) of the *I*_GABA_.

**Cmpd.**	**τ_des_/τ_des control_ 10 μM of steroid**	***P*-value**	***n***	**I_peak_/I_peak control_ 50 μM of steroid**	***P*-value**	***n***
1	0.99 ± 0.02	0.8273	5	0.86 ± 0.03	0.0040	8
2	0.98 ± 0.02	0.4625	6	0.77 ± 0.02	0.0001	7
3	0.98 ± 0.02	0.5264	6	0.95 ± 0.03	0.2608	6
4	0.80 ± 0.03	0.0014	6	0.75 ± 0.03	0.0001	7
5	0.97 ± 0.03	0.4228	6	1.12 ± 0.08	0.2069	8
6	0.99 ± 0.02	0.8273	5	0.92 ± 0.04	0.0926	7
7	0.98 ± 0.02	0.4626	5	0.77 ± 0.06	0.0055	7
8	0.88 ± 0.05	0.0265	6	0.75 ± 0.04	0.0005	8
9	0.97 ± 0.03	0.4228	5	0.99 ± 0.01	0.5690	8

**Mean ± SEM of the normalized values of the τ_*des*_ and I_*peak*_ of the I_*GABA*_ are shown. All comparisons with control value were made with unpaired Student’s t-test. Significance level of P = 0.05. n- the number of cells used.**

## Discussion

In the present study, we examined the effects of a series of endogenous NS on the GABA- and Gly-induced current in rat central neurons. It is known that NS modulate GABA_A_Rs and GlyRs functions in subunit-specific manner (Maksay et al., 2001; Belelli and Lambert, 2005) and this has implications for native receptors that may differentiate throughout development. We used in our experiments Wistar rats at 11–14 days of age where GlyRs and GABA_A_Rs were studied in pyramidal hippocampal neurons and cerebellar Purkinje cells, accordingly. Literature data indicate that starting from the second postnatal week, the subunit composition of GlyR in the hippocampal neurons (Aroeira et al., 2011) and GABA_A_R in the Purkinje cells of the cerebellum (Laurie et al., 1992) is close to that in the brain of adult animals. Extrasynaptic GlyRs with different subunit composition are described in pyramidal hippocampal neurons. There may be either heteromeric receptors with α (1, 2, or 3) and β subunits, or homomeric ones with multiple α subunits (for review, see Keck and White, 2009; Xu and Gong, 2010). The major adult isoform of GABA_A_Rs in Purkinje cells was shown to be composed of α_1_β_2_γ_2_ subunits and with a subunit stoichiometry of 2:2:1 (Pirker et al., 2000; Sieghart and Savić, 2018).

The series of steroids we studied included endogenous androstane and androstene NS (compounds **1**-**9**) with variable substituents at positions C-3, C-5, and C-17 (Table 1). In brief, compounds **1**, **3**, **7,** and **5** bear 17β- and 17α-hydroxyl groups, respectively. Compounds **2**, **4**, **6**, **8,** and **9** have a carbonyl group at C-17. Compounds **1**, **2**, **5**, **7**, and **8** have a double bond in their skeleton and as such belong to a family of androstene steroids. Oppositely, compounds **3**, **4**, **6** (5α-H), and **9** (5β-H) are fully saturated androstanes. The results of our study show that biological activity is similar for all compounds. In summary, compounds **1**-**9** at a concentration up to 10 μM strongly affected *I*_Gly_ and had weak action on *I*_GABA_. The effect of NS on *I*_Gly_ contained two components: a decrease in peak amplitude and an acceleration of decay. The effect of NS on *I*_Gly_ decay and the associated decrease in time constant of desensitization (τ_des_) was 2–3 times stronger than on the peak of *I*_Gly_. Such a different regulation of these two *I*_Gly_ parameters by NS suggests the existence of two independent mechanisms of their action on GlyRs, one of which regulates the peak amplitude, and the second – the desensitization process. This assumption is supported by our previous research (Bukanova et al., 2018), where it was shown that these two effects of NS afford different outcome with increasing glycine concentration. Namely, the effect on the peak amplitude of *I*_Gly_ disappeared and the acceleration of desensitization remained. The fact that peak inhibition is reduced at higher agonist concentration suggest that inhibiting drugs act as competitive inhibitors of agonist binding or that the inhibitors preferentially bind to resting states of the receptor (Li et al., 2007). However, the effect of NS on desensitization is insensitive to agonist concentration and therefore requires other explanations. In our opinion, the acceleration of the *I*_Gly_ decay can be explained by the slow block of the open channel or/and the acceleration of the desensitization gate (Gielen et al., 2015). Other authors (Borovska et al., 2012; Vyklicky et al., 2016) described the acceleration of the decay of NMDA current under the influence of NS and explain this effect by slow NS diffusion to the site of action at the extracellular vestibule of the NMDAR. At present, we cannot give preference to any of these assumptions regarding the mechanisms for accelerating the desensitization of *I*_Gly_ under the influence of NS. This remains to be elucidated.

Interestingly, in the literature, we have not found any indications of the ability of NS to accelerate the desensitization of *I*_Gly_. The published studies of the action of steroids on *I*_Gly_ were performed on recombinant GlyRs expressed in frog oocytes (Maksay et al., 2001), a chicken spinal neuron culture (Wu et al., 1990), and a rat hippocampal and spinal neuron culture (Jiang et al., 2009). In all of the described models available in the literature, the authors describe a decrease in the *I*_Gly_ peak amplitude under the influence of NS. The reason for this contradiction may be due to the features of the methodological approach. We use short (600–1000 ms) co-application of glycine and NS, while other authors used 10–30 s pre-application of the NS followed by 10–15 s application of glycine along with the NS. It is possible that the prolonged exposure of NS to the nerve cell leads to a change in properties of the structures responsible for the desensitization of the GlyRs. However, this issue requires special research. The IC_50_ values for the effect of compounds **1**-**9** on the τ_des_ of *I*_Gly_ were in the range of 0.12–0.49 μM, and on the peak amplitude – in the range of 16-22 μM. Our results are consistent with data from other authors who studied the effects of androsta(e)ne steroids with substitutions at C-17 on GlyRs. Maksay et al. (2001) showed that DHEA sulfate inhibits the recombinant GlyRs expressed in frog oocytes with an IC_50_ value of 2.5–6.3 μM.

As mentioned previously, GlyR-modulating compounds offer great potential for research on novel drug-like compounds. However, their parallel effect on GABA_A_R might be a disadvantage from the pharmacological perspective. Therefore, the discovery of a selective steroidal modulator of GlyR is a challenging task that has not been, according to our knowledge, described previously in the literature. Here, we demonstrate that the addition of compounds **3**, **5**, **6**, and **9** at a concentration of 0.1–50 μM did not change either the peak amplitude or the rate of desensitization of *I*_GABA_ in isolated Purkinje cells. In contrast, compounds **1**, **2**, **4**, **7**, and **8** at 50 μM concentration showed an inhibitory effect with a decrease in the peak amplitude of the current by 14–25% (*P* <0.01 or *P* < 0.05) and the acceleration of its desensitization by 23–45% (*P* <0.01). We conclude that compounds **3**, **5**, **6**, and **9** are selective modulators of *I*_Gly_. Their structures, however, do bear similar structural features to those that were able to affect *I*_GABA_. Therefore, establishing a pharmacophore from these results would be highly speculative. The data from the literature clearly indicate that a combination of C-3 and C-5 stereochemistry or the presence of double bond (4-ene/5-ene) of a steroid skeleton direct the effect on GlyRs and GABA_A_Rs activity (Park-Chung et al., 1999; Maksay et al., 2001; Fodor et al., 2006). Unfortunately, a simple additive approach cannot define pharmacophore for the desired combination of activity on one or both receptors. It is important to highlight that saturated 5α-H and unsaturated (4-ene/5-ene) steroidal skeletons possess a planar shape of the molecule, while the 5β-H skeleton is a “bent” structure. The global shape of the molecule is then significantly affected by the stereochemistry of the C-3 substituent. Note, that the 3α-hydroxy group of the planar 5α-H skeleton is axial, whereas the 3α-hydroxy group of the bent 5β-H skeleton is equatorial. Next, in case the substituent at C-3 is a carbonyl group, its location is in between axial and equatorial configuration. Finally, the nature of the modulatory effect seems to be defined by the substituent at position C-17. Taken together with the previously mentioned facts, we believe that we cannot define a pharmacophore for NS that would afford its modulatory action. Rather, a delicate balance of structural features at positions C-3, C-5, and C-17 could manage this extremely challenging task. The results of our unique study confirm this hypothesis. Our discovery of steroidal selective modulators of *I*_Gly_ provides a great potential for further structure-activity relationship studies affording novel compounds. Moreover, such research could lead to the identification of structural requirements of giving active compounds.

## Data Availability Statement

All datasets generated for this study are included in the article/supplementary material.

## Ethics Statement

The study was approved by the Ethics Committee of the Scientific Center of Neurology, Protocol No. 2-5/19 of 02.20.19.

## Author Contributions

JB conducted experiments to study the effects of neurosteroids on GABA- and glycine-activated current in rat neurons. ES wrote a physiological part of the manuscript. EK prepared compounds **6**-**9** as described in Materials and Methods section and wrote a chemical part of the manuscript.

## Conflict of Interest

The authors declare that the research was conducted in the absence of any commercial or financial relationships that could be construed as a potential conflict of interest.
